# Relative contributions from the ventricle and arterial tree to arterial pressure and its amplification: an experimental study

**DOI:** 10.1152/ajpheart.00844.2016

**Published:** 2017-06-03

**Authors:** Nicholas Gaddum, Jordi Alastruey, Phil Chowienczyk, Marcel C. M. Rutten, Patrick Segers, Tobias Schaeffter

**Affiliations:** ^1^Division of Imaging Sciences and Biomedical Engineering, King’s College London, St. Thomas' Hospital, London, United Kingdom;; ^2^King’s College London British Heart Foundation Centre, St. Thomas’ Hospital, London, United Kingdom;; ^3^Department of Biomedical Engineering, Technische Universiteit Eindhoven, Eindhoven, The Netherlands;; ^4^Ghent University, IBiTech-bioMMeda, iMinds Medical IT, Gent, Belgium; and; ^5^Physikalisch-Technische Bundesanstalt, Medical Physics and Metrological Information Technology, Berlin, Germany

**Keywords:** arterial pressure, hypertension, pulse pressure, pressure amplification, reflection, experimental model, arterial model

## Abstract

Arterial pressure is an important diagnostic parameter for cardiovascular disease. However, relative contributions of individual ventricular and arterial parameters in generating and augmenting pressure are not understood. Using a novel experimental arterial model, our aim was to characterize individual parameter contributions to arterial pressure and its amplification. A piston-driven ventricle provided programmable stroke profiles into various silicone arterial trees and a bovine aorta. Inotropy was varied in the ventricle, and arterial parameters modulated included wall thickness, taper and diameter, the presence of bifurcation, and a native aorta (bovine) versus silicone. Wave reflection at bifurcations was measured and compared with theory, varying parent-to-child tube diameter ratios, and branch angles. Intravascular pressure-tip wires and ultrasonic flow probes measured pressure and flow. Increasing ventricular inotropy independently augmented pressure amplification from 17% to 61% between the lower and higher systolic gradient stroke profiles in the silicone arterial network and from 10% to 32% in the bovine aorta. Amplification increased with presence of a bifurcation, decreasing wall thickness and vessel taper. Pulse pressure increased with increasing wall thickness (stiffness) and taper angle and decreasing diameter. Theoretical predictions of wave transmission through bifurcations werre similar to measurements (correlation: 0.91, *R*^2^ = 0.94) but underestimated wave reflection (correlation: 0.75, *R*^2^ = 0.94), indicating energy losses during mechanical wave reflection. This study offers the first comprehensive investigation of contributors to hypertensive pressure and its propagation throughout the arterial tree. Importantly, ventricular inotropy plays a crucial role in the amplification of peripheral pressure wave, which offers opportunity for noninvasive assessment of ventricular health.

**NEW & NOTEWORTHY** The present study distinguishes contributions from cardiac and arterial parameters to elevated blood pressure and pressure amplification. Most importantly, it offers the first evidence that ventricular inotropy, an indicator of ventricular function, is an independent determinant of pressure amplification and could be measured with such established devices such as the SphygmoCor.

## Abstract (precis)

The present study distinguishes contributions from cardiac and arterial parameters to elevated blood pressure and pressure amplification. Most importantly, it offers the first evidence that ventricular inotropy, an indicator of ventricular function, is an independent determinant of pressure amplification and could be measured with such established devices such as the SphygmoCor.

hypertension is one of the most important causes of global morbidity and mortality (16), and its prevalence in the developing world is rapidly increasing. The various contributors to high blood pressure are complex and not well understood beyond vascular resistance generating mean arterial pressure. Arterial stiffness, for example, has in more recent times been identified as contributing to pulse pressure (PP) (15, 23) and an important independent predictor of cardiovascular events (31). However, stiffness, indicated by the pulse wave velocity (PWV), is sensitive to both vessel geometry [thickness, diameter, taper (29)] and the wall’s material stiffness (Young’s modulus) and probably should therefore be understood as an “effective stiffness” of the vessel. Furthermore, pressure wave reflections have received substantial attention as an important contributor to pressure (20). Such reflections are created at changes in arterial impedance such as bifurcations (21), tapering (29), and changes in aortic stiffness (22), and its arrival back to the heart is signaled by the first systolic shoulder (19). However, it is difficult to isolate and quantify individual reflections; instead, they combine to create more of a “statistical notion” of a reflected wave (3).

Finally, the interaction between the ventricle and arterial impedance is an unquestionable contributor to arterial pressure (4). Clinically, the early systolic pressure gradient (dp/d*t*) is used as a marker for ventricular function as it is a surrogate for inotropy (26). However, it is difficult to decouple the interaction between the arterial impedance and ventricular stroke profile to characterize a patient’s pathology.

A practical understanding of arterial pressure contributions involves the discussion of arterial pressure evolution or amplification, as it is normally from peripheral measurement that one observes this load on the heart. Distal waveforms (typically brachial or femoral pressure), can too be analyzed as the sum of many Fourier components. The ratio of these Fourier components determine the amplification of certain frequencies and are indicative of the now popular pressure transfer function used to derive central pressure waveforms from distally measured ones (13, 17). Amplification has been associated with risk (12), increases with a higher heart rate (35) and male sex (27), and decreases with age (34). Like the contributors to pressure, however, the mechanisms affecting pressure amplification are not well understood (3) but have typically been associated with changes is arterial properties (6, 11, 25). Typically, amplification is up to 70% in young subjects (<20 yr old) and down to 20% in subjects over 80 yr old (18).

A significant predictive benefit has been observed measuring central pressure over the more convenient noninvasive brachial pressure (17). Interestingly, it was noted in the early 1990s that the transfer function between central and brachial pressure varies little with aging, allowing a generalized transfer function to be adopted for the general population specifically for deriving central from brachial pressure (13). Conversely, the transfer function from central to femoral artery (for example) has been shown to be highly variable (14, 20), most likely due to the long pulse transmission path through the age-affected aorta.

A deeper understanding of the mechanisms generating arterial pressure and affecting peripheral amplification toward more convenient measurement sites could accelerate developments in clinical diagnostics and therapy monitoring of hypertension. Numerical simulation of arterial networks has accelerated our understanding of the complex nature of pulse wave propagation in an arterial network, particular in the interaction of forward and backward propagating waves in aging and pathology (28, 30, 33, 36). Comparatively, however, there have been very few experimental models with which to validate these numerical studies. This is important when extrapolating the application of a validated model to investigate complex physiological phenomena; e.g., wave interaction through bifurcations and pulse propagating through biological arterial tissue versus materials with homogeneous mechanical models.

In this work, we developed an experimental cardiovascular simulator to examine the physiological responses to various changes in both ventricular and arterial parameters. Using this model, the aim of this study was to characterize the relative contributions of geometric and mechanical properties of the arteries, bifurcations, and the ventricular stroke profile on arterial pressure and the pressure amplification.

## METHODS

### 

#### Experimental setup.

**Fig. 1.**
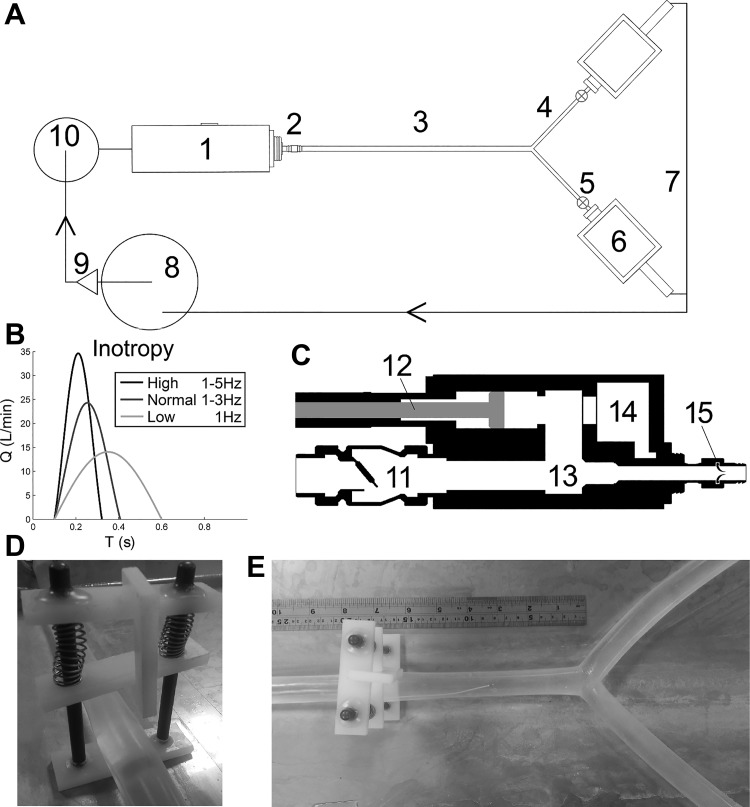
*A*–*E*: modular experimental rig schematic (*A*) with the ventricle side elevation (*C*) and programmed flow stroke profiles (*B*), the spring-loaded clamp for delivering impulse waves for the wave reflection analysis (*D*), and the spring-loaded clamp positioned to deliver an impulse to a silicone bifurcation model (*E*). The numbered items denote the following: *1*, the piston-driven ventricle; *2*, aortic valve; *3*, silicone arterial parent tube; *4*, silicone arterial child tube; *5*, Windkessel model; *6*, peripheral drain boxes; *7*, guttering system; *8*, lower pump; *9*, auxiliary pump; *10*, atrium; *11*, mitral valve; *12*, programmable stepper motor-driven ventricle piston; *13*, ventricle chamber; *14*, capillary chamber (not used in this study); and *15*, cross section of the aortic valve.

A piston/cylinder ventricle driven by a PC controlled (LabVIEW, National Instruments, Austin, TX) stepper motor (SMH60, Parker, Cleveland, OH) provided controllable and repeatable flow stroke profiles into various modular custom silicone arterial models (Fig. 1). Fluid entered the ventricle from the left atrium via a low vibration swing mitral valve [as used previously (7, 9)] and exited through a trileaflet-molded silicone aortic valve (LifeGroup, Eindhoven, The Netherlands). The working fluid was water.

Pressure was measured at two sites concurrently within the arterial models using intravascular pressure tip wires connected to a Millar pressure control unit (Mikro Cath 3.5F and PCU-2000, respectively, Millar, Houston, TX). With the use of these signals and the measured distance between the catheter tips, PWV was calculated using the foot-to-foot algorithm previously developed (8). Flow rate was measured using ultrasonic perivascular probes (PXL range to a T402 resolver, Transonic, Ithaca, NY). All signals were acquired at 1 kHz and processed retrospectively. Uniaxial stress/strain measurements (3340 Series, Instron, Norwood, MA) were acquired to determine the stiffness (Young’s modulus) of the arterial models.

The effect on arterial pressure and its amplification due to the ventricular stroke profiles and different arterial model geometries were observed. Fourier decomposition of the pressure waveform allowed analysis of the various frequency components.

#### Ventricular contribution to pressure.

Clinically, dp/d*t* is used as a marker for ventricular function as it is a surrogate for inotropy (26). To simulate varying inotropy experimentally, the time-varying position profile of the ventricle piston was programmed, so that the derivative of this profile multiplied by the area of the piston head provided a defined stroke profile in liters per minute (see Fig. 1, *B* and *C*). The Fourier representation of a sawtooth wave was used to recreate low inotropy, normal inotropy, and high inotropy by incrementally increasing the number of Fourier components used in the Fourier representation (32). With the use of just one component (a sine wave), a low systolic gradient and, therefore, low inotropy were generated. Increasing to three and then to five Fourier components, the piston stroke during systole becomes more rapid, inducing a more sawtoothed stroke profile, and, consequently, the systolic gradient increased. The three-component “normal inotropy” ejection profile has a systolic period (0.31 s) similar to that reported in the literature (10).

To compare simulations of different ventricular stroke profiles, the flow rate, mean arterial pressure, and heart rate were maintained at 3.8 l/min, 96 mmHg, and 60 beats/min, respectively. Stroke profile and heart rate (and therefore flow rate) were maintained by the software controlling the piston motion. Arterial pressure was generated using a manual screw valve at the end of each child vessel, so it could be controlled to create similarity in all tests. Maintaining the flow rate and heart rate meant that the diastolic period had to be varied (shorter with low inotropy and longer with high inotropy). Pressure and flow were monitored proximal to the aortic valve and at 400 mm distal to the valve.

#### Arterial parameter contributions to pressure.

The effects of vessel thickness (and therefore stiffness), diameter, and taper were investigated with the “normal” ejection input flow profile [Fig. 1, dark gray, with systolic time (*t*_sys_) = 0.31 s]. All arterial and ventricle parameters were tested with heart rates of 60 beats/min, in tubes of length (*L*) of 480 mm, and terminated with a 50-ml air volume compliance chamber and a clamp resistor modulated every test to provide a mean arterial pressure of 96 mmHg. Due to low diameters restricting the use of high flow, flow rates of 1.9 l/min were used for all tests. Silicone arterial models were created from either specialized molded tapering tubes (LifeGroup) or nontapering extruded silicone tube (Silco Products, Littlehampton, UK).

The contribution from wall stiffness was measured by using three tubes with wall thicknesses of 0.7, 1.0, and 1.5 mm and a diameter (Ø) of 20 mm. Pressure sensitivity to diameter was measured using two tubes with Ø = 20 mm in one tube and Ø = 15 mm in the other tube. To observe the effect of vessel taper four, different rates of taper were used, all with a thickness (*H*) = 0.8 mm. They each had a proximal inner diameters (Ø_1_) = 20 mm and distal diameters (Ø_2_) = 20, 15, 10, and 5 mm.

#### The bovine aorta and bifurcating model.

**Fig. 2.**
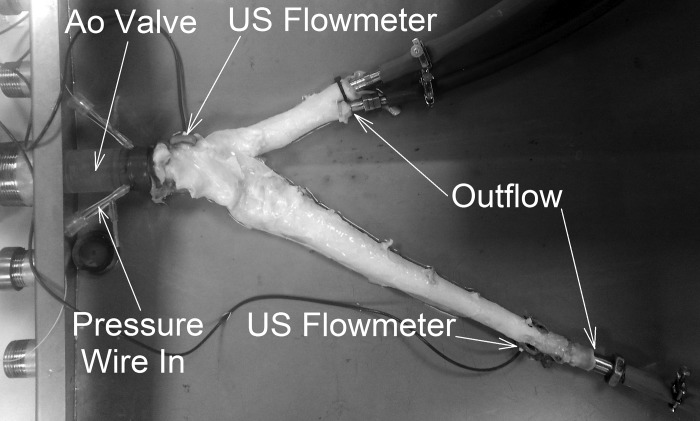
Bovine aorta (Ao) connected to the experimental model.

Following the basic characterization work described, the same analysis was carried out on a bovine aorta that was sourced from a local abattoir (see Fig. 2) as well as a physiologically relevant bifurcating silicon model (Fig. 1*E*).

The range of stroke profiles were applied to two arterials models. The silicone arterial model selected for this test is a tapering tube (Ø_1_ = 20 mm, Ø_2_ = 15 mm, *H* = 0.7 mm, and *L* = 480 mm), ending in a bifurcation with two 610-mm-long child vessel (Ø = 10 mm and *H* = 0.8 mm). The bovine aorta was transported chilled and then connected to the experimental model using water maintained at 37°C (thermostat-regulated heating system, A5B, ThermoFisher, Calsbad, CA) pumping through a heating coil immersed in the venous reservoir (Fig. 1, *top*, *number 8*). The bovine model included the proximal aorta (short section attached to the aortic valve, Ø = 33 mm, and *L* = 35 mm), a tapered upper body branch (Ø_1_ = 26 mm, Ø_2_ = 21 mm, and *L* = 106 mm) with two outflows, and a tapered lower body branch (Ø_1_ = 26 mm, Ø_2_ = 18 mm, and *L* = 375 mm) in which the distal pressure measurements were taken. The intercostal vessels were sealed each with a drop of cyanoacrylate.

#### Reflection and transmission from bifurcations.

Four bifurcation models were constructed using silicone tube varying the parent-to-child diameter ratio with parent tubes of Ø = 20 mm and child tubes of Ø = 20, 15, and 10 mm. Both symmetrical and asymmetrical configurations were constructed. Three symmetrical models were made with each child diameter variation and a consistent branching angle to the parent vessel axis of α = 40°. Finally, a nonsymmetrical model had a Ø = 20 mm parent with one child Ø = 15 mm with a branching angle of α = 0° (i.e., in line with the parent) and the other child Ø = 10 mm with a branching angle of α = 40°. All tubes had *L* = 400 mm and *H* = 0.8 mm.

A spring-loaded clamp (see Fig. 1, *D* and *E*) located 16 cm before the bifurcation created pressure impulses first originating in the parent tube and then in the child tube for each model (simulating pulse wave propagation from heart to the periphery and vice versa). Pull-back pressure wire measurements started 10 cm beyond the bifurcation and during successive impulses was sequentially withdrawn at 2-cm intervals back through the bifurcation toward the impulse clamp. A second pressure wire remained at the leading edge of the clamp itself to align the measurements in time. These measurements allowed the propagation of the impulse pressure peak to be monitored up the bifurcation, then its reflection as well as its transmission. Theoretical reflection (R_T_) and transmission (T_T_) coefficients, as given below,

(1)) $${\text{R}}_{\text{T}}=\frac{Y_{\text{P}}-(Y_{\text{C1}}+Y_{\text{C2}})}{Y_{\text{P}}+(Y_{\text{C1}}+Y_{\text{C2}})}$$

(2)) $${\text{T}}_{\text{T}}=1+{\text{R}}_{\text{T}}$$

were compared with those measured, where $Y=\frac{A}{\text{ρ}\;\times \text{ PWV}}$ is the tube admittance, *A* is the tube cross-sectional area, and ρ is fluid density (1).

**Fig. 3.**
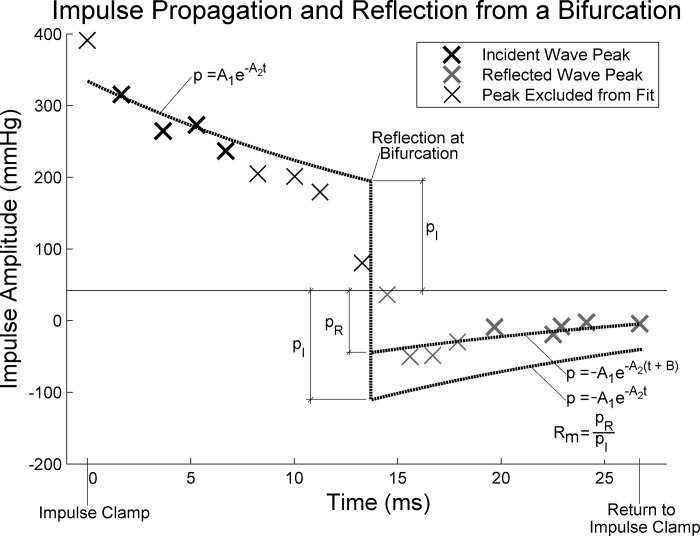
Tracking an impulse reflection; starting at a spring-loaded clamp [time (*t*) = 0 ms], propagating up to a bifurcation (*t* = 13 ms) and then returning back to the clamp (*t* = 26 ms).

Quantifying the measured reflection (R_M_) and transmission (T_M_) coefficients involved the fitting of an exponential decay curve to the peaks of the propagating incident impulse as well as the reflected/transmitted impulse and then comparing the relative amplitudes at the bifurcation site (see Fig. 3). The forward propagating incident and backward reflected waves overlapped and constructively interfered within ~10 cm of the bifurcation; therefore, these impulse peaks were excluded from analysis. Additionally, the impulse at the leading edge of the impulse clamp was excluded from analysis. This was because, as noted by Anliker et al. (2) in anesthetized dogs aortas, the amplitude of the exponential decay increases with frequency and therefore distorts any constant exponential decay.

An example analysis is shown in Fig. 3, where the peak of the propagating impulse can be seen decaying from the impulse clamp [just before time (*t*) = 0 s], down the tube toward the bifurcation (*t* = 13 ms), being reflected and then returning to the impulse clamp (*t* = 27 ms). The bold black and gray crosses in Fig. 3 indicate the incident and reflection peaks used in analysis, respectively. A first exponential decay function was fitted to the bold crosses between 0 and 13 ms and projected forward to the time of the pressure peak at the bifurcation. A second fictitious function was defined as the same as previous but negative, therefore having the same magnitude at the bifurcation at 13 ms. A third function was fitted to the bold crosses between 13 and 27 ms and then projected back to the bifurcation. The ratio of the magnitudes at the bifurcation of the second and third exponential projections defined R_M_ or, taking into account the transmitted impulse peaks, T_M_.

## RESULTS

### 

#### Material properties.

The stiffness of the bovine aorta from calculated as the mean measurement from the uniaxial Instron test from samples taken from the proximal aorta as well as just distal from the bifurcation and proximal to the outflow both on the tapered lower body branch. These measurements were done after the testing. The stiffness of the silicone tubes was also taken as the mean from a sample of three specimens cut from the tubing. These were 0.84 and 2.1 MPa, respectively.

#### Ventricular contribution to pressure.

**Fig. 4.**
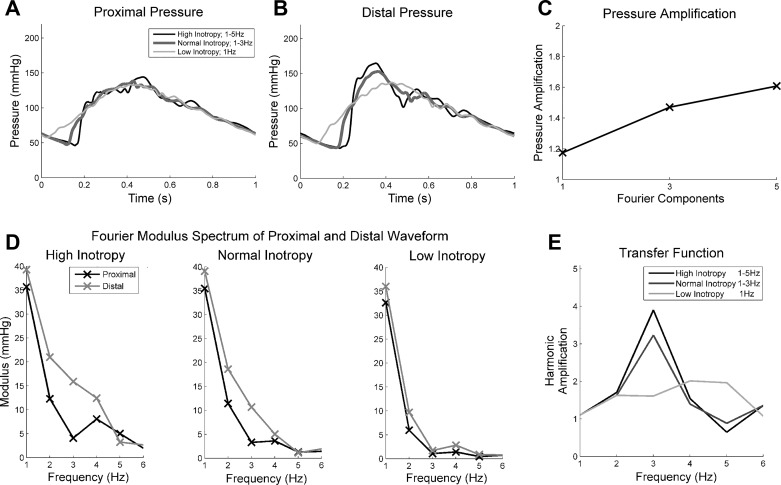
*A*–*E*: effect of varying inotropy. *A*–*C*: proximal pressure near the aortic valve (*A*), distal pressure (*B*), and pressure amplitude amplification over the first 40-cm distal to the aortic valve (*C*). *D* and *E*: moduli spectra of the first 6 Fourier components of pressure waveforms measured proximal to the valve and 400 mm distal to the valve in a tapering silicone artery with a bifurcation with a high, normal, and low inotropy ventricular flow profiles (*D*) and the resulting ratio of moduli or real part of the transfer function (*E*).

Although the PP was higher with the higher inotropy (Fig. 4, *A* and *B*), it should be noted that cardiac output was maintained between the ventricular contribution tests by changing the portions of systolic verus diastolic time. With higher inotropy, there was more time for the diastolic decay and therefore lower diastolic pressure. However, the systolic pressure remained constant between the tests (see Table 1). Most interesting, however, was that despite the arterial model not changing, the pressure amplification increased as the early systolic gradient increased (i.e., higher frequency components were included in the stroke profile). Quantitative comparisons are shown in Table 1.

**Table 1.** Ventricular and arterial contributions to arterial pressure and pressure amplification

	Proximal Pressure, mmHg			Distal Pressure, mmHg			
	Diastolic	Systolic	Pulse pressure	Diastolic	Systolic	Pulse pressure	Amplification
Ventricle							
Silicone							
High inotropy	52.47	147.92	95.46	46.35	176.88	130.53	1.61
Normal inotropy	55.90	140.49	84.60	51.25	159.39	108.14	1.47
Low inotropy	65.61	137.71	72.10	60.03	144.09	84.06	1.17
Bovine							
High inotropy	83.45	123.29	39.84	80.58	131.11	50.53	1.32
Normal inotropy	86.07	116.71	30.64	83.26	120.49	37.23	1.21
Low inotropy	87.87	113.52	25.66	84.69	115.04	30.34	1.10
							
Arterial							
Thickness							
0.5 mm	83.93	106.03	22.09	83.14	111.08	27.94	1.50
0.8 mm	60.16	120.74	60.58	63.78	134.47	70.70	1.28
1.5 mm	32.56	163.03	130.47	30.22	172.14	141.92	1.14
Taper							
20 to 5 mm	38.66	154.00	115.34	37.05	154.50	117.45	1.01
20 to 10 mm	50.85	134.18	83.34	48.95	139.50	90.55	1.09
20 to 15 mm	65.65	122.31	56.66	63.60	127.00	63.39	1.11
20 to 20 mm	69.00	111.75	46.55	67.25	121.75	54.50	1.24
Diameter							
15 mm	39.88	151.56	111.68	39.83	166.39	126.55	1.25
20 mm	60.43	121.71	61.28	634.78	135.40	71.62	1.27
							
Amplification per meter							
Before bifurcation, 130−440 mm	1.35						
Through bifurcation, 440−500 mm	2.10						
After bifurcation, 500−740 mm	1.10						

The waveforms were broken down into Fourier components to determine what was contributing to the increase in amplification. The proximal and distal Fourier modulus spectrum is shown for each of the three ventricular stroke profiles and the amplification of the Fourier moduli (or the real part of the transfer function, see Fig. 4*D*). As higher frequency contributions were incorporated (from low, to normal, to high inotropy), contributions from these higher frequency (2–5 Hz) were produced in the proximal pressure waveforms. As these proximal components of these higher frequencies increased, so too did the distal contributions and subsequently the pressure amplification increased. As the arterial model remains constant, one would expect a constant transfer function regardless of ventricular inotropy. However, as the moduli are so low for both the sinewave stroke profile above 2 Hz and normal stroke profile over 4 Hz, the resulting transfer functions above these upper limits are generated by noise only. With this in mind, the high inotropy transfer function was very similar to the normal transfer function between 1 and 4 Hz and the sine transfer function between 1 and 2 Hz (see Fig. 4*E*).

#### Arterial parameter contributions to pressure.

**Fig. 5.**
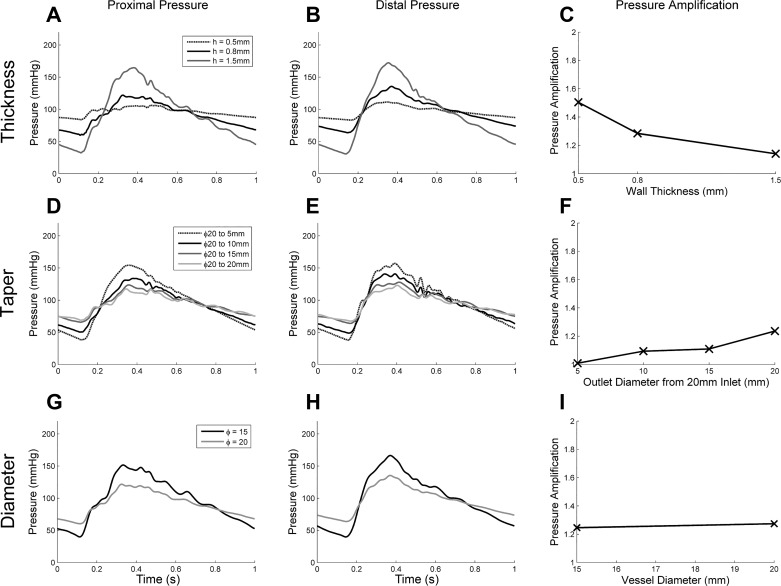
*A*–*I*: proximal pressure near the aortic valve (*A*, *D*, and *G*), distal pressure (*B*, *E*, and *H*), and pressure amplitude amplification over the first 40 cm distal to the aortic valve (*C*, *F*, and *I*). Wall thickness variation (*A*–*C*), varying degrees of taper (*D*–*F*), and varying vessel diameters (*G*–*I*) are shown.

Elevated PP was caused by increased thickness and taper and decreased diameter (see Fig. 5). Pressure amplification over the first 40 cm of the silicone aorta models increased with increasing vessel taper and decreased with increasing thickness. Very little change in amplification was observed with a changing diameter from 15 to 20 mm. Quantitative comparisons are shown in Table 1.

**Fig. 6.**
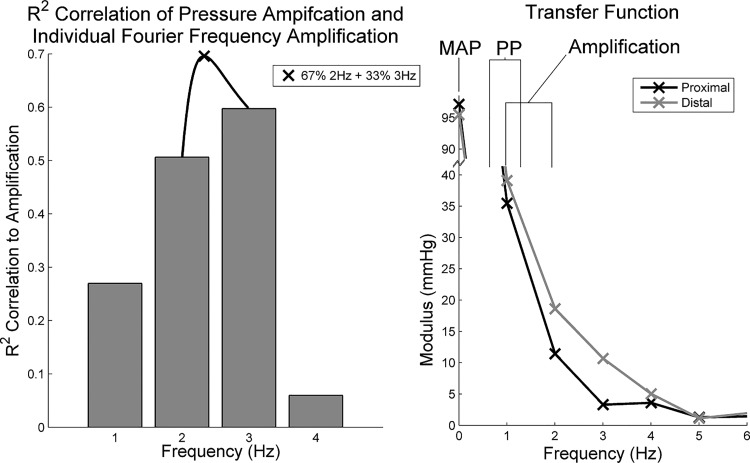
*Left*: analysis of which of the amplified frequencies correlate highest with peripheral pressure amplification. *Right*: transfer function map to indicate which frequencies contribute to mean arterial pressure (MAP), pulse pressure (PP), and amplification.

All geometry modulations cause variation in the transfer function. In particular, the geometries with higher harmonic amplifications between 2 and 3 Hz of the transfer function correlated with the higher pressure amplification. Further analysis (the black line between the 2- and 3-Hz bars in Fig. 6, *left*) showed that the highest correlation (*R*^2^ = 0.69) with pressure amplification was found with a 67% contribution from the 2 Hz combined with a 33% contribution from the 3-Hz Fourier components (Fig. 6, *left*). We can therefore generalize that higher contributions in the 2- to 3-Hz range will contribute to high pressure amplification, whereas elevated contributions around 1 Hz (at a normal heat rate) indicate a high PP (see Fig. 6, *right*).

#### The bovine aorta and bifurcating model.

**Fig. 7.**
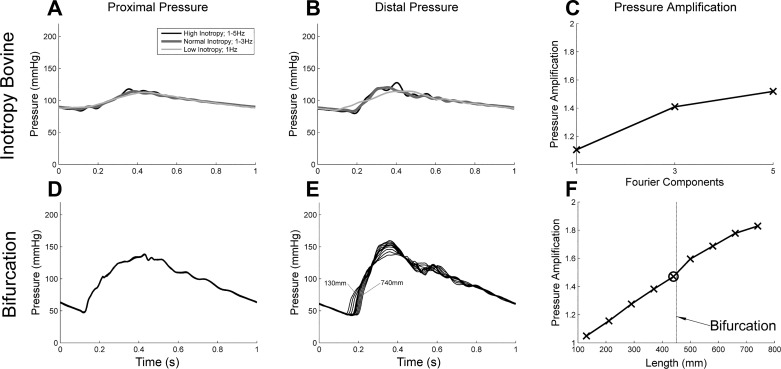
*A*–*F*: proximal pressure near the aortic valve (*A* and *D*), distal pressure (*B* and *E*), and pressure amplitude amplification (*C* and *F*). Ventricular inotropy variation in a bovine aorta (*A*–*C*), along the parent and then child vessels of a bifurcating silicon arterial tree (*D*–*F*). Note the development of the distal pressure wave from 130 mm distal of the aortic valve, to the bifurcation at 480 mm (bold profile), and then to 740 mm from the aortic valve down one of the child vessels.

The inotropy variation study shown in Fig. 4 was repeated in a bovine aorta and a more physiologically realistic silicone model (tapered aorta with femoral bifurcation). Applying the normal ventricular stroke profile, the PP measured in the silicone tube (Fig. 4, *A*–*C*) was substantially higher than in the bovine aorta (84.6 vs. 30.64 mmHg, respectively), (see Fig. 7, *A*–*C*). This is likely due to the higher stiffness of the silicone contributing to a lower total compliance of the system.

Amplification was also affected by a bifurcation. A nonlinearity in the rate of amplification along the arterial model was observed (see the gradient change indicated at the location of the bifurcation, Fig. 7, *D*–*F*, and quantified in Table 1), which to the authors’ knowledge has not been documented before. In the parent tube, the amplification rate was 1.35 m^−1^, which increased to 2.10 m^−1^ at the bifurcation and then back to 1.10 m^−1^ in the child tube.

#### Reflection and transmission from bifurcations.

**Fig. 8.**
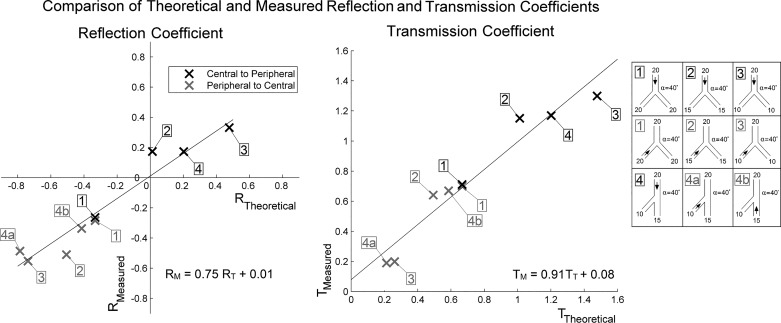
Comparison of theoretical and measured reflection (*left*) and transmission (*right*) coefficients measured in four bifurcating tube configurations using an impulse created in both the parent (black) and child (gray) tubes. R_M_, reflection coefficient; T_M_, transmission coefficient.

Both the measured reflection (R_M_) and transmission (T_M_) ratios were lower than those predicted theoretically using *Eqs. 1* and *2* (see Fig. 8). However, the transmission ratio had a correlation closer to unity than the reflection ratio, 0.91 (*R*^2^ = 0.94) and 0.75 (*R*^2^ = 0.94), respectively. Symmetry did not appear to affect the trend indicating that branch angle may not play an important role in modulating the reflection between α = 0 and 40°. Transmission back from the periphery was significantly lower than transmission forward from the parent vessel, supporting claims of the bulk of reflections becoming trapped in the peripheral vessels (5).

To ensure the impulse propagation was comparable to normal physiology, impulse PWVs were measured in all three tube diameters and compared with theory according to the measured stiffness of the silicone and tube diameters [Branwell Hill equation (15)]. The wave speeds in the 20- and 15-mm tubes were equivalent to the theory to one decimal place (9.3 and 10.7 m/s, respectively). There was higher variation in the 10-mm tube with a measured wave speed of 13.1 m/s compared with a theoretical wave speed of 11.9 m/s.

## DISCUSSION

We constructed an experimental cardiovascular model including a programmable ventricular stroke profile, various arterial models, and venous return to investigate important contributions to arterial pressure. The specific aim of this study was to characterize the relative contributions of geometric and mechanical properties of the arteries, bifurcations, and the ventricular stroke profile on arterial pressure and pressure amplification. Comparisons were made by maintaining the flow rate, mean arterial pressure, and heart rate constant at 3.8 l/min, 96 mmHg, and 60 beats/min for all tests. The experimental model allowed ventricular and arterial parameters to be modulated in isolation to observe their distinct contributions to the pressure wave and its propagation.

### 

#### Contributions from the ventricle.

Ventricular inotropy was modulated by changing the shape of the ventricular stroke profile by changing the number of Fourier components comprising a theoretical sawtooth wave, between one (low inotropy) and five (high inotropy) components (Fig. 1*B*). Incorporation of higher frequencies increased systolic pressure gradient and peak flow and decreased the systolic time. However, it appears as though the difference in PP was only due to the increased portion of the cardiac cycle spent during diastole, and inotropy therefore probably does not affect PP (see Fig. 4).

Contrary to published data (6, 11), higher inotropy had a more remarkable impact upon pressure amplification than any of the arterial parameters within the ranges tested in this study. To the authors’ knowledge, this has not been previously described but can be explained simply by breaking the propagating pressure wave into Fourier components. Statistical analysis indicated that pressure amplification was most sensitive to increases in the 2- to 3-Hz contributions, which is determined by the early systolic gradient of the input flow wave. This corroborates with clinical observations that dp/d*t* is used as a surrogate for inotropy and indicator of ventricular function (26).

The silicone model saw an amplification over 400 mm of 61% and 17% when the high and low inotropy ejection profiles were used, respectively (see Table 1). These reflect clinical observation of 70% in subjects under 20 yr old and 20% in subjects over 80 yr old (18). This trend was repeated with the bovine aorta, although with lower PP and amplitudes of 32% and 10%, respectively, due to the lower stiffness of the aortic tissue (0.84 MPa compared with 2.10 MPa for the silicone).

#### Contributions from arteries.

The effect on proximal pressure in our experimental results echo those that have been presented in numerical studies (28, 30, 33), where vessel wall thickness, diameter, and taper each affected the “effective stiffness” and, subsequently, PP generated proximal to the valve. The lower the diameter, whether a straight tube or tapered, the less material available over which to distribute strain in response to the distending pressure (see Fig. 5). Subsequently, lower diameters make the vessel appear stiffer. Similarly, increasing the wall thickness simulated stiffening of the silicone material itself, also increasing PP.

This study has also offered insights into the role of the ventricle in pressure generation and its amplification (Fig. 4*D*). Variation in arterial properties modulates the shape of the transfer function when measured along the aorta and consequently the amplification of the more prominent lower frequency (2–3 Hz) components of the pressure wave. Additionally, a nonlinearity in the rate of amplification along the vessels was also observed at the bifurcation. In the parent tube, the amplification rate was 1.35 m^−1^, which increased to 2.10 m^−1^ at the bifurcation and then back to 1.10 m^−1^ in the child tube (see Table 1). This effect has not been reported before and indicates the value in maintaining experimental validation studies. Lower wall thickness (stiffness) and reduced taper increased the pressure amplification along the vessel. This reflects observations of higher pressure amplification in younger populations [low stiffness and lower proximal aortic taper angles (24)] compared with older subjects.

The simplicity of this model allowed for individual contributions to be isolated, quantified, and then modulated. Future work should develop this mode into a more physiologically representative model to capture the transition from central elastic arteries to stiffer peripheral arteries. Furthermore, capturing the native arterial tree’s geometry would allow the measures to be transferable to the clinic.

It should be noted that increased thickness was used as surrogate for increased stiffness. This is reasonable when the Young’s modulus of the material is unchanged, as it was in this study. Of course, increased thickness can be associated with both an increase in material stiffness (such as inflammation or calcification) as well as a lower material stiffness, such as in Ehlers-Danlos syndrome.

#### Contributions from bifurcations.

Despite lower than theoretically predicted reflections/transmissions, reflected pressure waves from tapering and bifurcating vessels provide important contributions to arterial pressure. With high “effective” stiffness of the vessels, the resulting earlier arrival of the statistical peak of the reflected wave has an important impact on the PP.

Low variations were seen in the linear regression fits for both the reflection and transmission correlations (*R*^2^ = 0.94 for both correlations). The correlation of the theoretical and measured transmitted wave amplitudes through a silicone bifurcation was 0.91, indicating theory (1) predicts quite well. Reflection, on the other hand, had a poorer correlation of 0.75, indicating some losses in mechanically reflecting the incident wave.

As arteries (whether silicone or tissue) are flexible tubes with viscous components, it is reasonable that efficiency considerations should be applied to the analysis of wave propagation. Certainly, in our mechanical model, the bifurcations oscillated axially and radially whether impulse or physiological waves are propagated through them. These oscillations are extensions and compressions of the silicone walls, which would account for at least some of the energy absorption. This suggests a positive developmental trait for humans as the bifurcations reduces the energy of the central pressure augmenting reflected wave while allowing pressure energy to be transmitted and alter trapped in the periphery. Nevertheless, further work is needed to account for the efficiencies observed and to where the remaining 25% of reflection energy is dissipated. These observations can be directly applied to numerical simulations of the cardiovascular system to better capture the mechanics of wave propagation through the arterial tree.

#### Limitations.

For the bulk of this study, both water and silicone were used as analogs for blood and the human aorta and large arteries. Where possible, comparisons to clinical measurements were provided in the results. However, quantities provided should be used for comparative analyses of the contributors to pressure and amplification only. The ventricular stroke profile investigations in the bovine aorta showed that tissue arteries have a significantly lower effective stiffness than the silicone tubes used, in this case due to lower material stiffness and larger proximal aorta diameter. Despite this, the physical laws that govern pressure generation and propagation in the human arterial system are transferable to flexible arterial networks with higher or lower effective stiffness.

The validity of silicone models to represent wave reflection/transmission of arterial tissue is important to consider. In terms of the pressure distension relationship of silicone, this of course differs from human arteries in that it does not simulate the composite elastic/collagen response. However, provided the pressures are sufficiently low as to not engage the collagen, then the elastic response of silicone should be a reasonable representation of the elastic response of elastin. From a reflection point of view, Anliker et al. (2) published two studies in the late 1960s where mechanical vibrations were applied to the thoracic aorta of anesthetized dogs. In these studies, they also observed amplitude decay in the pressure signal of ~50% over the first 10 cm. This decay was very similar to the decay we observed, as shown in Fig. 3. In this case, the pressure decayed from ~400 to 250 mmHg, with a baseline pressure of ~50 mmHg, which indicates a decay of ~45% in the 0.8-mm-thick silicone tubes.

#### Clinical application.

These results can be translated to diagnosis of the cause of hypertensive pressure (i.e., discerning ventricular pathology from arterial pathology) as well as the design of vascular devices. This is of particular interest in the use of β-blockers to explain observations of their ability to reduce pressure amplification (3). Hypertension is likely a symptom of various combinations of a number of static (arterial geometry/stiffness) and dynamic (ventricular contraction) pathologies. Treatment normally involves the prescription of single or combination drug therapies. If pressure is not reduced, then quantities or the drugs themselves are varied until they are pharmacologically managed. One obvious application of these data could be to observe amplification from carotid to brachial arteries, affected little by aging, as an indication of the contribution from ventricular inotropy, thus offering cheap, pressure sensor-based ventricular assessment. Another could be to investigate transfer functions across other vessel paths such as carotid to femoral artery to see if distinguishing trends can be seen between patients with stiff aortas, as opposed to high peripheral resistance. Practically, this could be realized with a device such as the SphygmoCor. Interestingly, this device has become the gold standard for aortic PWV measurement, providing the opportunity to distinguish contributions from arterial stiffness and ventricular inotropy within the same measurement. Further clinical study is needed to fully evaluate the utility of this approach.

#### Conclusions.

Contributions to arterial pressure generation and peripheral amplification from various ventricular and arterial parameters were investigated in a novel experimental model. In contrast to published literature claiming that arterial stiffness is the primary modulator of pressure amplification, our results reveal that in fact the ventricle is a substantial contributor to amplification. Pressure amplification was elevated by increased ventricular systolic gradient induced by higher contributions from higher frequencies in the ventricle’s stroke profile. To the authors’ knowledge, this is the first time the ventricle has been independently linked to this critical parameter, which may offer noninvasive diagnostic opportunity. Furthermore, pressure amplification was affected by the presence of a bifurcation, was higher when the arteries were thinner (less stiff) and with less taper. PP was augmented by arterial parameters that increased effective stiffness, such as wall thickness, reduced diameter, and increasing taper. Although theoretical transmission predictions were close to those measured, pressure wave reflection had an efficiency of only 75% indicating significant energy losses in reflection not accounted for by theory. The study allows the first comparison of contributions to arterial pressure from many of the parameters viewed as important for hypertension.

## GRANTS

N. Gaddum, J. Alastruey, and S. Schaeffter acknowledge funding from the Engineering and Physical Sciences Research Council (EPSRC; Project Grant EP/K031546/1), the Centre of Excellence in Medical Engineering (funded by the Wellcome Trust and EPSRC under Grant WT 088641/Z/09/Z), support from the National Institute for Health Research (NIHR) Biomedical Research Centre award to Guy's and St Thomas' National Health Service (NHS) Foundation Trust in partnership with King's College London, and the NIHR Healthcare Technology Co-operative for Cardiovascular Disease at Guy’s and St Thomas’ NHS Foundation Trust. The views expressed are those of the authors and not necessarily those of the NHS, NIHR, or Department of Health.

## DISCLOSURES

No conflicts of interest, financial or otherwise, are declared by the author(s).

## AUTHOR CONTRIBUTIONS

N.G., M.C.R., P.S., and T.S. conceived and designed research; N.G. performed experiments; N.G. analyzed data; N.G., J.A., P.S., and T.S. interpreted results of experiments; N.G. prepared figures; N.G. drafted manuscript; N.G., J.A., P.C., M.C.R., P.S., and T.S. edited and revised manuscript; N.G., J.A., P.S., and T.S. approved final version of manuscript.
